# Mechanisms Linking the Gut-Muscle Axis With Muscle Protein Metabolism and Anabolic Resistance: Implications for Older Adults at Risk of Sarcopenia

**DOI:** 10.3389/fphys.2021.770455

**Published:** 2021-10-26

**Authors:** Konstantinos Prokopidis, Edward Chambers, Mary Ni Lochlainn, Oliver C. Witard

**Affiliations:** ^1^Department of Musculoskeletal Biology, Institute of Life Course and Medical Sciences, University of Liverpool, Liverpool, United Kingdom; ^2^Department of Metabolism, Digestion and Reproduction, Faculty of Medicine, Imperial College, London, United Kingdom; ^3^Department of Twin Research and Genetic Epidemiology, King’s College London, London, United Kingdom; ^4^Faculty of Life Sciences and Medicine, Centre for Human and Applied Physiological Sciences, King’s College London, London, United Kingdom

**Keywords:** anabolic resistance, sarcopenia, gut microbiota, dietary fiber, skeletal muscle, exercise

## Abstract

Aging is associated with a decline in skeletal muscle mass and function—termed sarcopenia—as mediated, in part, by muscle anabolic resistance. This metabolic phenomenon describes the impaired response of muscle protein synthesis (MPS) to the provision of dietary amino acids and practice of resistance-based exercise. Recent observations highlight the gut-muscle axis as a physiological target for combatting anabolic resistance and reducing risk of sarcopenia. Experimental studies, primarily conducted in animal models of aging, suggest a mechanistic link between the gut microbiota and muscle atrophy, mediated via the modulation of systemic amino acid availability and low-grade inflammation that are both physiological factors known to underpin anabolic resistance. Moreover, *in vivo* and *in vitro* studies demonstrate the action of specific gut bacteria (*Lactobacillus* and *Bifidobacterium*) to increase systemic amino acid availability and elicit an anti-inflammatory response in the intestinal lumen. Prospective lifestyle approaches that target the gut-muscle axis have recently been examined in the context of mitigating sarcopenia risk. These approaches include increasing dietary fiber intake that promotes the growth and development of gut bacteria, thus enhancing the production of short-chain fatty acids (SCFA) (acetate, propionate, and butyrate). Prebiotic/probiotic/symbiotic supplementation also generates SCFA and may mitigate low-grade inflammation in older adults via modulation of the gut microbiota. Preliminary evidence also highlights the role of exercise in increasing the production of SCFA. Accordingly, lifestyle approaches that combine diets rich in fiber and probiotic supplementation with exercise training may serve to produce SCFA and increase microbial diversity, and thus may target the gut-muscle axis in mitigating anabolic resistance in older adults. Future mechanistic studies are warranted to establish the direct physiological action of distinct gut microbiota phenotypes on amino acid utilization and the postprandial stimulation of muscle protein synthesis in older adults.

## Introduction

Sarcopenia is described as the age-related decline in skeletal muscle mass and function (Rosenberg, 1997) that was recently recognized as an independent geriatric condition (Cao and Morley, 2016) and is reported to affect 8–13% of older adults (Shafiee et al., 2017). Although aging is associated with a progressive decline in muscle mass and strength, an accelerated deterioration of muscle functional capacity has been observed in individuals with sarcopenia (Cruz-Jentoft et al., 2019). The clinical implications of sarcopenia include—but are not limited to—an increased incidence of falls and fractures, frailty, loss of mobility and independence, and premature mortality among older adults (Cruz-Jentoft et al., 2019). Hence, understanding the interplay between physiological mechanisms that underpin sarcopenia is fundamental to developing targeted and effective lifestyle approaches to reduce sarcopenia risk in our aging population.

Multiple physiological factors are proposed to underpin sarcopenia. These factors include—but are not limited to—age-related changes in hormonal milieu (Sakuma and Yamaguchi, 2012), and gut physiology (Azzolino et al., 2019), a chronic state of low-grade inflammation (Beyer et al., 2012), insulin resistance (Cleasby et al., 2016), DNA damage, elevated oxidative stress, mitochondrial dysfunction (Jackson and McArdle, 2016), and suppressed satellite cell activity (Snijders et al., 2015), as reviewed previously (von Haehling et al., 2012). Ultimately, muscle atrophy is underpinned by a state of negative muscle protein balance whereby rates of muscle protein breakdown (MPB) exceed rates of muscle protein synthesis (MPS) over a given period of time. Of these two metabolic processes, there is general consensus that a diminished capacity for older adults to stimulate MPS, as opposed to an acceleration of MPB, mediates muscle atrophy with aging, at least in healthy individuals (Tipton et al., 2018). In this regard, whereas comparative studies of young and older adults have reported no clear differences in basal postabsorptive rates of MPS, an impaired response of MPS to ingestion of meal-like quantities of protein (Guillet et al., 2004; Cuthbertson et al., 2005; Moore et al., 2015) and/or other anabolic stimuli such as resistance exercise (Kumar et al., 2009; Durham et al., 2010) have been consistently reported with advanced age. This metabolic phenomenon has been coined muscle anabolic resistance (AR) and is proposed to contribute to the progressive decline in skeletal muscle mass associated with aging.

The physiological mechanisms that mediate AR are multi-factorial but, to this end, are not fully understood (Burd et al., 2012). Fundamentally, the diminished capacity for older adults to stimulate MPS is underpinned by a reduced systemic (Bohé et al., 2003) and/or intracellular availability of amino acids (Kimball and Jefferson, 2002). Physiological processes that contribute to this age-related decline in amino acid availability include an increased splanchnic retention of amino acids leading to reduced peripheral amino acid availability (Boirie et al., 1997), a reduction in amino acid transport to muscle tissue (Biolo et al., 1995), and an impairment in microvascular perfusion (capillary recruitment and dilation) (Phillips et al., 2015). Recent evidence also indicates an important role for the human gut microbiota environment in regulating the utilization of amino acids (Yatsunenko et al., 2012; Ticinesi et al., 2019). In this regard, gut microbiota dysbiosis (Mahnic et al., 2020) is a physiological phenomenon that describes an altered gut microbiota composition (Biagi et al., 2010; Odamaki et al., 2016) and diversity (Nagpal et al., 2018; Coman and Cristian, 2020), and is proposed as another mediator of age-related AR. Further evidence also exists that an altered gut microbiota may directly increase risk of sarcopenia through specific bacterial depletion and fecal transplantation (Liu et al., 2021). Hence, the aims of this opinion narrative review are two-fold. First, to offer hypothesis-driven insights into possible pathophysiological mechanisms linking gut microbiota dysbiosis with impaired skeletal muscle metabolism in older adults. Second, and based on limited existing evidence, to propose a series of potential, non-pharmacological, strategies targeted at combatting AR via modulation of the gut microbiota. Interventional approaches addressed in this narrative review are by no means exhaustive and are focused on dietary fiber consumption, probiotic and prebiotic supplementation, and resistance exercise training.

## Gut Microbiota in Aging

The structure and diversity of the human gut microbiome plays a key regulatory role in physiological, metabolic, and immune function, and thus impacts human health and disease risk (Guinane and Cotter, 2013). Specifically, the gut microbiome contains millions of diverse microorganisms, termed gut microbiota, that modulate various metabolic pathways, including inflammatory gene expression, innate immune effector cells (i.e., monocytes, macrophages), glucose tolerance, and the release of gut hormones (Martin et al., 2019; Zheng et al., 2020). The multiple microbial phyla of the gut microbiome include *Proteobacteria, Fusobacteria, Actinobacteria, Verrucomicrobia*, *Firmicutes* (*Clostridium, Enterococcus, Ruminococcus, Lactobacillus*), and *Bacteroidetes* (*Prevotella, Bacteroides*), and account for the majority of the microbiota species present in the gut (Wang et al., 2020), although several bacterial species may be found in other organs including muscle, brain, liver, heart, and adipose tissue (Lluch et al., 2015).

The composition of gut microbiota is modulated by several factors including genetics, diet and physical activity levels (Milani et al., 2016; Ticinesi et al., 2017). Aging also is strongly associated with a decline in gut microbiome diversity species in the duodenum, jejunum, ileum, and colon (Sovran et al., 2019; Badal et al., 2020). Taxonomic differences during aging have been observed, namely that older adults are accompanied by higher levels of *Bacteroides, Eubacterium*, and *Clostridiaceae*, and decreased *Bifidobacterium* compared to young adults (Yatsunenko et al., 2012; Odamaki et al., 2016; Stewart et al., 2018). During the aging process, epithelial cell tight junctions are weakened (Lustgarten, 2016), decreasing the expression of intestinal epithelial tight junctions proteins (Tran and Greenwood-Van Meerveld, 2013). This disrupted intestinal barrier is linked to reduced intestinal motility and increased permeability, that are associated with higher levels of low-grade inflammation and immunosenescence that accompany various age-associated conditions (Belkaid and Hand, 2014; Bosco and Noti, 2021). Therefore, the various taxonomic changes that occur over time via altered microbial function and composition may affect immune and metabolic health with advancing age (Ling et al., 2020).

## The Gut-Muscle Axis in Sarcopenia

Multiple lines of evidence from rodent studies suggest that the gut microbiota may be linked with sarcopenia. First, the microbiota of older mice was shown to exhibit an abundance of the *Rikenellaceae* family that is associated with an increased frailty index in a dose-dependent manner (Langille et al., 2014). Second, a higher *Sutterella* to *Barneseilla* ratio has been reported in older sarcopenic vs. healthy adult rats, corresponding with an altered inflammatory and immune profile and decline in triceps and gastrocnemius size (Siddharth et al., 2017). Third, germ-free mice that lack the gut microbiota of pathogen-free mice exhibit a greater decline in skeletal muscle mass, quality and neuromuscular function compared to pathogen-free mice (Lahiri et al., 2019), despite having similar body weight (Hsu et al., 2015). Finally, antibiotic-treated mice were accompanied by increased muscle atrophy (Manickam et al., 2018; Nay et al., 2019; Okamoto et al., 2019), that was associated with microbial dysbiosis and inhibition of ileal fibroblast growth factor 15 (FGF15), whereas muscle atrophy was reversed following FGF19 treatment (Qiu et al., 2021). Hence, some mechanistic evidence exists in animal models that the gut microbiome may play a key role in physical performance, given that germ-free and antibiotic-treated mice express lower competence during muscle loading (Lahiri et al., 2019) and swimming time to exhaustion (Hsu et al., 2015; Huang et al., 2019), compared to pathogen-free mice.

The hypothesis that the gut microbiota may be linked with sarcopenia has also been examined in humans. Using 16s RNA sequencing, a higher abundance of *Lactobacillus* and a reduction of *Fusicantenibacter, Eubacterium, Lachnospira, Lachnoclostridium*, and *Roseburia* genera was reported in sarcopenic patients compared with healthy controls (Kang et al., 2021). In addition, cross-sectional studies have revealed a higher ratio of *Firmicutes/Bacteoidetes* and lower overall microbial richness in older adult patient groups compared with healthy young adult controls (Mariat et al., 2009; Larsen et al., 2010; Claesson et al., 2012; Le Chatelier et al., 2013). Accordingly, a higher abundance of several bacteria, including *Eggerthella*, *Bacteroides/Prevotella, Lactobacillus/Enterococcus*, and a lower abundance of *Enterobacteriaceae*, *Methanobrevibacter*, and *Akkermansia*, have been observed in frail patient groups (Van Tongeren et al., 2005; Ponziani et al., 2021). Moreover, in sarcopenic and physically frail populations, an increased abundance of *Oscillospira* and *Ruminococcus*, and a decrease of *Barnesiellacaea* and *Christensenellaceae* taxa also have been reported (Picca et al., 2020). Similarly, sarcopenic patients displayed a significant reduction in *Faecalibacterium prausnitzii, Roseburia inulinivorans*, and *Alistipes shahii* species that are all competent bacteria with prominent metabolic capacity in producing SCFA (Ticinesi et al., 2020). The 16S rRNA sequencing of human fecal samples from (pre)sarcopenic individuals showed a decline in *Lachnospira, Fusicantenibacter, Roseburia, Eubacterium*, and *Lachnostrodium* genera, and an increased LPS biosynthesis compared to healthy individuals (Kang et al., 2021). Taken together, these data indicate a link between the gut microbiota and muscle atrophy, and thus supports a gut-muscle axis hypothesis to explain, in part, skeletal muscle dysfunction during aging.

## Gut Microbiota and Anabolic Resistance

**FIGURE 1:**
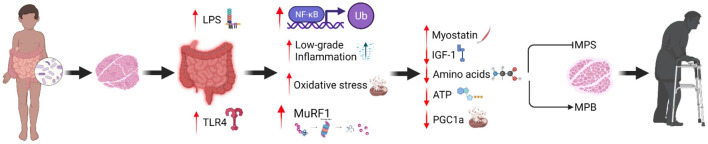
Proposed mechanisms that underpin muscle anabolic resistance via changes in gut microbiota diversity.

The gut microbiota also is proposed to play a causal role in AR (Frampton et al., 2020), as mediated by multiple inter-related physiological mechanisms (Figure 1). The causal mechanisms that underpin AR span several levels of physiology, including the gut, vascular system, and skeletal muscle (Burd et al., 2013). Altered gut microbiota composition during aging may be involved in oxidative stress, inflammation, and insulin resistance (Grosicki et al., 2018). An aging gut microbiota may increase intestinal permeability and LPS leakage from the intestinal lumen and cell membranes of gram-negative bacteria into the circulation (Maes et al., 2012; Alhmoud et al., 2019). Such modifications are associated with insulin resistance and increased inflammation (Liang et al., 2013; Choi et al., 2015; Moszak et al., 2020), both of which are physiological factors linked with increased risk of sarcopenia (Nelke et al., 2019; Shou et al., 2020). Older adults are characterized by increased LPS levels that enhance toll-like receptor 4 (TLR4) signaling, promoting metabolic endotoxemia (Ghosh et al., 2015). Metabolic endotoxemia may induce systemic inflammation through reactive oxygen species production (Yuan et al., 2013) and activation of apoptotic pathways (i.e., nuclear factor κB, c-Jun N-terminal kinase), downregulating immune function in older adults (Qian et al., 2012). Specifically, proinflammatory cytokines (i.e., IL-6 and TNF-a) may modulate LPS-induced proinflammatory responses through TLR4/Mal signaling pathway (Greenhill et al., 2011). Therefore, the altered function and composition of gut metabolites during aging may be responsible for metabolic perturbations that develop throughout lifespan.

Regarding gut physiology, amino acid absorption is modulated at the cellular level via active transport by the epithelial intestinal cells in the small intestine located at the surface of enterocytes (Mardinoglu et al., 2015). These transporters shuttle amino acids into the circulation (Broer, 2008) via the peptide transporter 1 carrier (Walther et al., 2019). Recent evidence implicates a role for the human microbial environment in amino acid homeostasis (Yatsunenko et al., 2012; Ticinesi et al., 2019). Specifically, human studies demonstrate that the microbial-derived amino acids, e.g., threonine and lysine, are incorporated into the free plasma amino acid pool following consumption of a moderate protein diet, suggesting that disruption of the gut microbiota environment may suppress the microbial-induced production of amino acids (Metges et al., 1999) and potentially lead to AR. Furthermore, during dietary protein restriction, the gut microbiota is reported to produce amino acids through *de novo* biosynthesis (Lin et al., 2017) and is implicated in amino acid homeostasis via FGF21 hepatic signaling (Martin et al., 2021). However, the role of the gut microbiome in modulating the uptake of amino acids, in particular the branched-chain amino acids, into the skeletal muscle cell is yet to be fully elucidated in humans. Future studies are warranted to fill this gap in knowledge, with the likely focus on leucine uptake by skeletal muscle given the role of leucine as both a substrate and signal for the stimulation of MPS (Anthony et al., 2001).

An alternative mechanism linking the gut microbiota with AR relates to insulin-like growth factor 1 (IGF-1). IGF-1 synthesis regulates nutrient sensing and the stimulation of MPS (Bian et al., 2020) that is modulated, in part, by the gut microbiota (Yan and Charles, 2018). This notion is supported by previous studies that associated circulating levels of IGF-1 with a decrease in *Salmonella typhimurium* and *Burkholderia thailandensis* infected mice compared to *Escherichia coli O21: H+* treated mice that retained IGF-1/Akt pathway capacity (Schieber et al., 2015). At the mechanistic level, IGF-1 is regulatory for muscle growth via the phosphatidylinositol 3-kinase (PI3K)/Akt signaling pathway, and serves to suppress the mRNA transcription and translation process of MPS (Barclay et al., 2019). A recent study that utilized a 16S ribosomal RNA gene sequencing approach revealed the gut microbiota of intestinal epithelial cell-specific IGF-1 knockout mice exhibited a disrupted intestinal homeostasis and epithelial regeneration in comparison to mice under normal pathological conditions (Zheng et al., 2018). Hence, it has been suggested that intestinal permeability due to microbiota dysbiosis may induce systemic inflammation (Soendergaard et al., 2017; Thevaranjan et al., 2017) and suppress IGF-1R sensitivity, thus initiating a catabolic response through MuRF-1 expression (Barclay et al., 2019). Given the potential physiological role of the gut microbiota in regulating skeletal muscle metabolism via the modulation of amino acid homeostasis and/or IGF-1 activity, a current focus of physiology research into healthy musculoskeletal aging relates to optimizing the gut microbiota (Ticinesi et al., 2019; Ni Lochlainn et al., 2021). It may be considered intuitive that lifestyle (i.e., physical activity, exercise, and diet) approaches targeted at modulating the microbial environment may mitigate AR associated with sarcopenia.

## Microbiome-Centric Dietary Strategies to Counter Anabolic Resistance

Microbial activity induced by indigestible amino acids promote the production of metabolic end products including short-chain fatty acids (SCFA; acetate, butyrate, propionate), branched-chain fatty acids (BCFA; valerate, isobutyrate, isovalerate), ammines, phenols, thiols, indoles, and ammonia. SCFA modulate epithelial cell function and microbiome physiology, serving as the primary energy source of colonocytes, and thus influence gastrointestinal health (Canfora et al., 2015). Specifically, acetate is utilized by skeletal muscle cells for ATP production, whereas the metabolic fate of butyrate and propionate primarily relates to gluconeogenesis and cholesterol synthesis (Byrne et al., 2015). SCFA are produced by dietary fiber fermentation (i.e., resistance starch, oligofructose, inulin, polydextrose, galactoolisaccharides) in the colon and are absorbed via the portal vein during lipid digestion (Chambers et al., 2018b). In addition, bacterial cross-feeding modulates SCFA production and substrate utilization with regards to human gut physiology (Ríos-Covián et al., 2016; Tsukuda and Yahagi, 2021). For instance, co-cultured *Bacteroides uniformis* and *Escherichia coli* were more effective in agarooligosaccharide degradation compared to their isolated properties (Li et al., 2014). Similarly, *Bifidobacterium adolescentis* co-cultured with *Bifidobacterium infantis* and *Roseburia A2-183* strains exhibited a synergistic effect on agarotriose utilization and butyrate production (Belenguer et al., 2006; Li et al., 2014). Hence, cross-feeding of bacteria taxa is a primary contributor of SCFA synthesis and utilization that may provide useful insight in designing future microbiome-centric interventions to counter AR.

**FIGURE 2:**
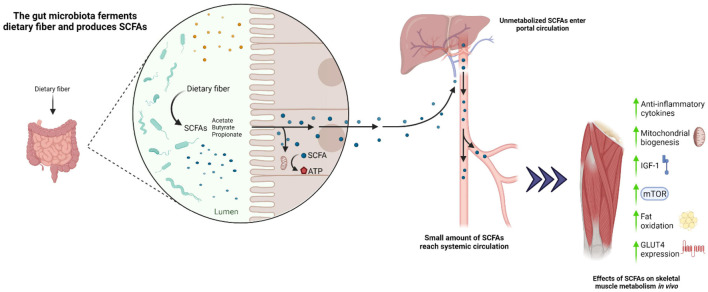
Proposed role of the gut microbiota in modulating skeletal muscle metabolism under conditions of increased dietary fiber.

Several amino acids, including glycine, threonine, glutamate, lysine, arginine, ornithine, and aspartate, also play an important role in acetate production, whereas threonine, lysine, and glutamate are involved in butyrate synthesis, and threonine is involved in propionate synthesis (Davila et al., 2013). SCFA are increasingly recognized as modulators of skeletal muscle metabolism via action of the G protein-coupled receptors GRP41 (FFAR3) and GRP43 (FFAR2) (Nilsson et al., 2003; Kimura et al., 2014). GPR41 and GRP43 are understood to stimulate GLP-1 and PYY secretion and increase insulin-mediated glucose uptake in skeletal muscle (Canfora et al., 2015). *In vivo* studies have demonstrated improvements in insulin sensitivity, mitochondrial biogenesis and function, reduced adiposity, and an increase in type I muscle fiber composition following SCFA administration (Fushimi et al., 2001; Gao et al., 2009; Hong et al., 2016; Zhou et al., 2021) that correspond with an increased myoglobin expression (Yamashita et al., 2014; Maruta et al., 2016; Figure 2). Accordingly, germ-free mice supplemented with SCFA were shown to exhibit greater muscle (gastrocnemius) mass and strength compared to germ-free controls (Lahiri et al., 2019). Likewise, a 10-week butyrate-enriched diet improved mitochondrial biogenesis, insulin sensitivity, and muscle (quadriceps and gastrocnemius) mass in aged mice compared to butyrate-free controls, whereas no distinguishable differences were observed between younger groups (Walsh et al., 2015). Collectively, these data imply that SCFA administration may be beneficial in mitigating AR in mice. Likewise, in a human study of young and older adults, the administration of butyrate and propionate was shown to improve fat oxidation, insulin sensitivity, and inflammatory profiles (Chambers et al., 2015, 2018a, 2019; Cleophas et al., 2019). Furthermore, an increased capacity for gut microbial synthesis of butyrate was associated with elevated *Faecalibacterium prausnitzii* and *Butyricimonas virosa*, and higher skeletal muscle index (Lv et al., 2021). Interestingly, older adults with a higher dietary fiber density (grams of fiber consumed/100 kcal) displayed a positive relationship with increased whole body lean mass and butyrate-producing bacteria, including *Ruminococcus, Lachnospira*, and *Clostridia* (Barger et al., 2020)] compared with older adults consuming lower fiber intake. Consistent with this observation, 13 weeks of soluble fiber supplementation in older adults led to improvements in handgrip strength, although changes in gut microbiota and SCFA were not examined in this study (Buigues et al., 2016). These observations are in line with a recent study, highlighting an association of higher dietary fiber intake with increased skeletal muscle mass and strength in middle-aged to older adults (Frampton et al., 2021). To date, no clinical studies have investigated the direct impact of SCFA and high soluble fiber diets on skeletal muscle protein metabolism. Hence, future studies are warranted to investigate the chronic impact of manipulating dietary fiber content in promoting gut derived SCFA and modulating skeletal muscle metabolism and inflammation in older adults.

## Prebiotic and Probiotic Supplementation as a Strategy to Counter Anabolic Resistance

Preliminary evidence, based on a limited number of hypothesis-driven studies, suggests that probiotic supplementation may confer physiological benefits to host physiology bacteria and thus promote skeletal muscle anabolism. Several studies have investigated the impact of *Lactobacilli* administration on metabolic function. Specifically, there is evidence that *Lactobacillus plantarum* may exert muscle anabolic effects by enhancing protein assimilation and upregulating the activation of mTOR as a molecular driver of MPS, as demonstrated using Drosophila models (Storelli et al., 2011; Erkosar et al., 2015). This observation may be explained, at least in part, by a shift in gut microbiota to a higher abundance of butyrate-producing species, leading to an increased IGF-1 activity and reduced pro-inflammatory cytokine secretion, as observed in *Lactobacillus* supplemented mice compared to germ-free counterparts (Bindels et al., 2012; Chen et al., 2016, 2020). Furthermore, administration of *Lactobacillus paracasei PS23* resulted in greater mitochondrial function and reduced inflammatory cytokine activity in senescence-accelerated mice, with potential implications for reducing sarcopenia risk (Chen et al., 2019). Consistent with this finding, the administration of probiotics containing *Lactobacilli* species was shown to reduce systemic levels of IL-6 and TNF-a (Barreto et al., 2014; Borzabadi et al., 2018), and improve amino acid absorption kinetics in humans (Jäger et al., 2020). Hence, there is physiological rationale, albeit relatively limited experiential evidence, to suggest that multiple *Lactobacillus* strains may mitigate AR through a concomitant decrease in systemic inflammation and a greater amino acid utilization in the gut.

The administration of multiple bacterial species may be another strategy to target the gut microbiota and counteract AR. Microbiota transplantation from healthy or undernourished infants into young germ-free mice has demonstrated an increased accumulation of *Ruminococcus gnavus* and *Clostridium symbiosum* that ameliorated lean body mass gains and muscle growth (Blanton et al., 2016). Consistent with this observation, the transfer of gut microbiota from lean vs. obese pigs to germ-free coincided with marked increases in gastrocnemius muscle fiber size (Yan et al., 2016), thus highlighting the potential impact of administering multiple bacteria through nutritional targets (i.e., synbiotic supplementation).

The combination of prebiotics (non-digestible fiber) and probiotics, collectively termed synbiotics, provides an emerging nutritional strategy to ingest non-digestible fiber in order to promote the development of specific gut microbiota species. Synbiotics consist of *Bifidobacterium* and *Lactobacillus* species and have been shown to reduce lipid accumulation, enhance muscle performance, and improve gut barrier function in aged mice (Ni et al., 2019). Moreover, synbiotics may suppress low-grade inflammation through SCFA administration, mediated via a greater composition of colonic bacteria communities in older adults (Lahtinen et al., 2009; Macfarlane et al., 2013), although this observation is not universal (Neto et al., 2013). Accordingly, probiotic (i.e., *Lactobacillus salivarius, Lactobacillus plantarum TWK10*) and prebiotic (i.e., inulin) supplementation has been proposed as a promising strategy to facilitate the provision of a healthy gut microbiota, reducing systemic inflammation and improving exercise performance and muscle strength (Xiao et al., 2014; Chen et al., 2019; Lee et al., 2020). Consistent with this notion, several experimental trials have demonstrated microbial enrichment accompanied by reduced proinflammatory cytokine secretion (Vulevic et al., 2008), improved insulin sensitivity (Cani et al., 2009; van der Beek et al., 2018), handgrip strength (Buigues et al., 2016) and frailty conditions (Theou et al., 2019), and increased SCFA concentrations (Rebello et al., 2016) in both young and older adults. Hence, various parameters (i.e., low-grade inflammation and insulin resistance) associated with AR may be attenuated with prebiotic, probiotic, and/or synbiotic administration. To our knowledge, no studies have investigated the impact of microbial species on the stimulation of MPS and/or activation of mTOR related signaling in older adults. Therefore, future studies are warranted to investigate the impact of prebiotic or probiotic supplementation on the production of gut microbiota strains and subsequent stimulation and/or suppression of MPS and MPB, respectively, in young and older adults.

## Exercise as a Strategy to Counter Anabolic Resistance Via the Modulation of Gut Microbiota

Exercise/physical activity is a crucial component of any strategy designed to prevent and/or treat sarcopenia, which may be mediated, in part, by modulating the gut microbiome (Strasser et al., 2021). Accordingly, the impact of exercise training in modulating the gut microbiota has primarily been established in physically active populations and animal models in the context of aerobic-based exercise (Cerdá et al., 2016; Taniguchi et al., 2018; Morita et al., 2019; Przewłócka et al., 2020; Clauss et al., 2021). Specifically, *Bacteroides fragilis* gnotobiotic mice improved swimming exercise capacity and reduced physical fatigue compared to germ-free mice (Hsu et al., 2015), and similar results were observed in gnotobiotic models containing *Eubacterium rectale, Lactobacillus plantarum*, and *Clostridium coccoides* bacteria (Huang et al., 2019). Similarly, inoculation of *Veillonella atypica* in mice improved running exercise capacity and appeared to be mediated via the conversion of exercise-induced lactate to propionate (Scheiman et al., 2019), whereas the combination of aerobic exercise with *Bifidobacterium longum* administration may further improve aerobic capacity and inflammatory status as demonstrated in mice supplemented with a probiotic strain isolated from an elite Olympic athlete (Huang et al., 2020). Recently, Valentino et al. (2021) showed that antibiotic-treated mice resulted in a disrupted gut microbiome, which was correlated with less hypertrophy of soleus type I and IIa, and plantaris type IIb muscle fibers compared to untreated counterparts following progressive weighted wheel running (Valentino et al., 2021). Furthermore, another study characterized professional athletes as exhibiting more diverse microbial communities and bacterial species involved in SCFA production compared to age-matched sedentary individuals (Barton et al., 2018). This observation may explain the role of exercise training in promoting SCFA biosynthesis (Clarke et al., 2014; Dumas et al., 2017; Murtaza et al., 2019). Consistent with this observation, the implementation of a training program that combined aerobic and resistance exercise resulted in an increased abundance of *Blautia*, *Dialister*, and *Roseburia*, and decreased abundance of *Proteobacteria* and *Gammaproteobacteria* phylum in obese children (Quiroga et al., 2020). However, 12 weeks into the intervention, no differences in gut microbiota profile were detected between the obese children and an age-matched, healthy control cohort.

A bidirectional relationship between exercise and bacterial strains has been reported in older adults supplemented with *Lactobacillus casei*, which was associated with increased physical activity levels as measured via daily step count (Aoyagi et al., 2019). Observational data reveal that physically active older adults are characterized by an increased abundance of *Bifidobacteriales* and *Clostridiales* species (Castro-Mejía et al., 2020). This observation is not consistent with previous studies that indicated a decreased microbial diversity during sedentarism (Bressa et al., 2017; Castellanos et al., 2020). Moreover, differences in bacteria taxa have been observed through higher *Faecalibacterium prausnitzii* and lower *Parasutterella excrementihominis* between physically active and community-dwelling older adults (Fart et al., 2020). However, limited data in humans has been generated regarding the modulation of gut microbiota with resistance training in older adults.

Although endurance exercise confers multiple metabolic health benefits, including improved insulin sensitivity, mitochondrial function, and maximal oxygen consumption (Bird and Hawley, 2017; Hargreaves and Spriet, 2020), resistance training provides the most robust anabolic stimulus to mitigate age-related AR (Mcleod et al., 2019). Preliminary *in vivo* data suggest an improvement in gut microbiota diversity and composition in response to resistance training (Chen et al., 2021). Specifically, a previous study revealed a reduced relative abundance of pro-inflammatory-induced species, including *Pseudomonas*, *Serratia*, *Comamonas*, and *Firmicutes/Bacteroidetes* ratio that translated to a decrease in intestinal mucosal permeability and enriched SCFA-producing gut microbiota. Whereas no changes in the gut microbiome with resistance training were observed in young adults (Bycura et al., 2021), this study may be considered to lack statistical power in terms of microbiome sampling, and the short duration of intervention (8 week) implemented may have been insufficient to elicit detectable changes in the gut microbiome (Bycura et al., 2021). Interestingly, a seminal study by Fielding et al. (2019) revealed that transferring fecal samples from high-functioning older adults to mice increased the abundance of *Barnesiella intestinihominis* bacteria that corresponded with improved grip strength compared with low-functioning-colonized mice (Fielding et al., 2019). Indeed, the high-functioning-colonized mice displayed a higher number of *Prevotellaceae* family, *Prevotella* and *Barnesiella* genus, and *Barnesiella intestinihominis* species compared to low-functioning-colonized mice. These findings align with previous work linking an elevated *Prevotellaceae* family profile to young professional athletes (Clarke et al., 2014), and *Prevotella* and *Barnesiella* to less frail phenotypes (Van Tongeren et al., 2005; Claesson et al., 2012; Verdi et al., 2018). Accordingly, these data suggest that these microbes are associated with better physical conditioning. Moreover, genes derived from *Barnesiella* and *Prevotellaceae* may produce SCFAs, that could further explain the improved muscle functional outcomes germ-free mice following a SCFA cocktail (Lahiri et al., 2019). Taken together, these preliminary findings indicate that resistance exercise may lead to the production of SCFA metabolites, contributing to improved muscle strength, although this thesis warrants direct investigation in older adults. Future studies designed to examine the impact of resistance training on bacteria taxa and SCFA production and their influence on the gut microbiome in young and older adults would provide more reliable conclusions in humans.

## Conclusion

Preliminary evidence, based on a limited number of hypothesis-driven studies primarily conducted using animal models, suggests a mechanistic action for the gut microbiota in countering age-related AR and sarcopenia risk. However, at present, clinical trials are warranted to validate these microbial-induced outcomes on skeletal muscle using *in vivo* human models. Based on findings from animal- and cell-based models, there is evidence to suggest that improvements in *de novo* amino acid biosynthesis may correspond with the maintenance of gut microbiota diversity through promotion of SCFA via dietary fiber and protein consumption. Moreover, *in vivo* experiments have demonstrated an increased SCFA production following exercise that may attenuate AR by enhancing amino acid utilization and reducing levels of low-grade inflammation. Moving forward, the design of lifestyle approaches that combine increased dietary fiber and protein intake, probiotic supplementation and resistance training may be effective in optimizing gut microbiota composition with implications for muscle health in older adults at risk of sarcopenia.

## Author Contributions

KP and OW conceived and wrote the initial draft of the manuscript. EC and MN reviewed and revised the manuscript. All authors contributed to the article and approved the submitted version.

## Conflict of Interest

The authors declare that the research was conducted in the absence of any commercial or financial relationships that could be construed as a potential conflict of interest.

## Publisher’s Note

All claims expressed in this article are solely those of the authors and do not necessarily represent those of their affiliated organizations, or those of the publisher, the editors and the reviewers. Any product that may be evaluated in this article, or claim that may be made by its manufacturer, is not guaranteed or endorsed by the publisher.
